# Luminescent and Magnetic Tb-MOF Flakes Deposited on Silicon

**DOI:** 10.3390/molecules26185503

**Published:** 2021-09-10

**Authors:** Elena Bartolomé, Ana Arauzo, Sergio Herce, Anna Palau, Narcis Mestres, Sara Fuertes, Pablo Sevilla, Nicholas S. Settineri, Laura Navarro-Spreafico, Jonay González, E. Carolina Sañudo

**Affiliations:** 1 Department of Mechanical Engineering, Escola Universitària Salesiana de Sarrià (EUSS), Passeig de Sant Joan Bosco 74, 08017 Barcelona, Spain; psevilla@euss.es; 2 Instituto de Nanociencia y Materiales de Aragón (INMA), CSIC-Universidad de Zaragoza, 50009 Zaragoza, Spain; aarauzo@unizar.es; 3 Institut de Ciència de Materials de Barcelona (ICMAB-CSIC), 08017 Barcelona, Spain; sherce@icmab.es (S.H.); palau@icmab.es (A.P.); narciss@icmab.es (N.M.); 4 Departamento de Química Inorgánica, Facultad de Ciencias, Instituto de Síntesis Química y Catalisis, Homogénea (ISQCH), CSIC-Universidad de Zaragoza, 50009 Zaragoza, Spain; sfuertes@unizar.es; 5 Advanced Light Source, Lawrence Berkeley National Laboratory, Berkeley, CA 94720, USA; nsettine@berkeley.edu; 6 Department of Chemistry, University of California, Berkeley, Berkeley, CA 94720, USA; 7 Secció de Química Inorgànica, Departament de Química Inorgànica i Orgànica Universitat de Barcelona, C/Martí i Franquès, 1-11, 08028 Barcelona, Spain; laura.spre@gmail.com (L.N.-S.); jgonzasa22@alumnes.ub.edu (J.G.); 8 Institut de Nanociència i Nanotecnologia, IN2UB, C/Martí i Franqués 1-11, Universitat de Barcelona, 08028 Barcelona, Spain

**Keywords:** 2D MOFs, lanthanides, luminescence, magnetic anisotropy, exfoliation, flakes

## Abstract

The synthesis of a terbium-based 2D metal–organic framework (MOF), of formula [Tb(MeCOO)(PhCOO)_2_] (**1**), a crystalline material formed by neutral nanosheets held together by Van der Waals interactions, is presented. The material can be easily exfoliated by sonication and deposited onto different substrates. Uniform distributions of Tb-2D MOF flakes onto silicon were obtained by spin-coating. We report the luminescent and magnetic properties of the deposited flakes compared with those of the bulk. Complex **1** is luminescent in the visible and has a sizeable quantum yield of QY = 61% upon excitation at 280 nm. Photoluminescence measurements performed using a micro-Raman set up allowed us to characterize the luminescent spectra of individual flakes on silicon. Magnetization measurements of flakes-on-silicon with the applied magnetic field in-plane and out-of-plane display anisotropy. Ac susceptibility measurements show that **1** in bulk exhibits field-induced slow relaxation of the magnetization through two relaxation paths and the slowest one, with a relaxation time of *τ*_lf_ ≈ 0.5 s, is assigned to a direct process mechanism. The reported exfoliation of lanthanide 2D-MOFs onto substrates is an attractive approach for the development of multifunctional materials and devices for different applications.

## 1. Introduction

Two-dimensional (2D) materials are receiving growing interest owing to their huge potential for application in electronics [1], energy storage [2,3,4], catalysis [5], sensing, biomedicine [6], etc., and the intriguing new physics appearing at the 2D limit [7,8].

While most well-studied 2D materials are inorganic solids, such as the very well-known graphene [9], recent advances in coordination chemistry have enabled the synthesis of 2D metal–organic frameworks (2D MOFs), which can be functionalized in different ways and thus represent a flexible and versatile alternative [10]. The large horizontal and ultra-thin dimensions of 2D MOFs lead to very high values of specific surface, atomic surface ratio and number of active places exposed to surface, which can enhance some capabilities when compared to bulky materials [11,12]. There are already many examples of 2D MOFs, usually relying on polytopic planar ligands or molecules that afford a coordination network in two dimensions with a suitable linker [13,14].

The study of magnetic MOFs based on transition metal (3d) and lanthanide (4f) ions is of particular interest. Different types of magnetic MOFs are being investigated [15], such as materials with magnetic cooperativity, spin-crossover [16], magnetocaloric effect [17], and MOFs with slow magnetic relaxation [18], where the nodes are either single-ion magnets (SIMs) [18], single-molecule magnets (SMMs) [19], or which embed single-chain magnets (SCMs) [20].

Another interesting possibility concerns the development of multifunctional complexes that incorporate multiple properties such as magnetism and luminescence [21,22,23,24,25,26,27,28] or photochromism [29,30].

The preparation of 2D molecular layers on surfaces is a prerequisite for the development of many applications. Different approaches have been utilized for this purpose, such as the self-assembly of evaporated molecules [31], the grafting of molecules on functionalized surfaces [32,33], and the use of surface-coordinated MOF thin films (SURMOFs) [34]. The use of hard-templates has also been proposed as an interesting strategy for engineering the structure of MOFs onto surfaces, as recently reviewed in [35].

For 2D MOFs formed of neutral Van der Waals nanosheets, exfoliation by sonication in a liquid matrix has been shown to produce nanosheets stable towards aggregation [36], [37]. However, the isolation of high-quality flakes of 2D MOFs in large amounts that can be suitably deposited onto surfaces remains a challenge. The preparation of high-quality crystalline layers with lateral sizes of 8 μm and thicknesses of 4 nm of a whole family of Fe-based magnetic MOFs, through a liquid exfoliation procedure was recently presented by Coronado et al. [38].

In a recent work, we presented a facile, microwave-assisted method for the preparation of a dysprosium carboxylate-based 2D MOF material, that could be exfoliated into nanosheets by sonication [39].

Herein, we used this microwave-assisted method to synthesize the terbium 2D MOF analogue, of formula [Tb(MeCOO)(PhCOO)_2_] (**1**), formed by the stacking of neutral nanosheets held together only by Van der Waals interactions. The material can be exfoliated by sonication and deposited onto different substrates by spin-coating. We report the luminescent and magnetic properties of **1** in the form of flakes deposited onto silicon wafers, compared to those of the bulk counterpart.

## 2. Results

### 2.1. Synthesis and Crystal Structure

The synthesis of [Tb(MeCOO)(PhCOO)_2_]_n_ (**1**) takes place in a microwave reactor. A microwave pulse applied to the reagents in 1:1 MeOH:MeCN heats the reaction mixture homogenously and favors one intermediate reaction, thus leading to a pure product. After 10 min, the reaction mixture is cooled down and a small amount of solid is obtained. This precipitate is [Tb(MeCOO)(PhCOO)_2_] (**1**). The solid is then separated and the solution is left in a laboratory oven at 40 °C for one to two weeks; after this time, colorless crystals of **1** can be filtered. At room temperature, larger crystals but in smaller quantities are obtained within similar periods of time. The crystal structure of **1** is obtained using synchrotron radiation due to the very small size of the crystals. Table 1 shows the crystallographic parameters for **1**.

**Figure 1:**
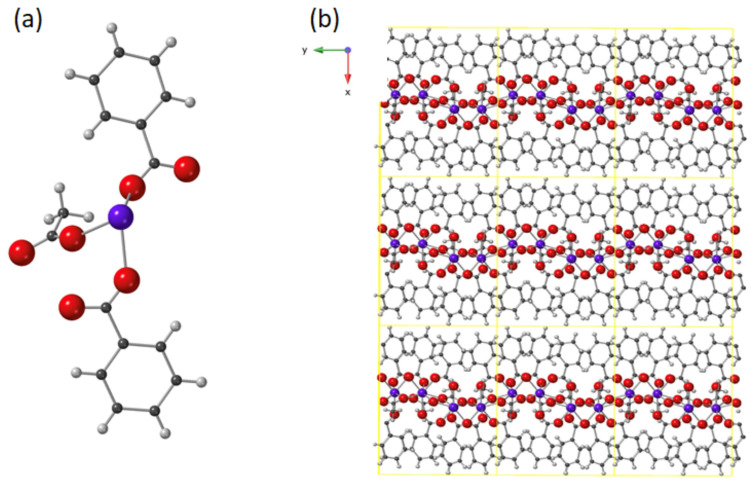
(**a**) Asymmetric unit of **1**, [Tb(MeCOO)(PhCOO)_2_], and (**b**) crystal packing showing three Van der Waals stacked layers of **1**. Unit cell shown in yellow. Tb: purple; Oxygen: red; Carbon: dark grey; H: light grey.

Figure 1 shows the asymmetric unit of compound **1** and the crystal packing. Compound **1** is isostructural to the Ln=Dy compound earlier described [39]. The Tb(III) ion has coordination number 8, with a distorted square antiprism coordination environment like the Dy analogue. The crystal structure consists of Van der Waals stacked nanosheets of 2D MOFs of formula [Tb(MeCOO)(PhCOO)_2_]_n_, as shown in Figure 1b.

**Figure 2:**
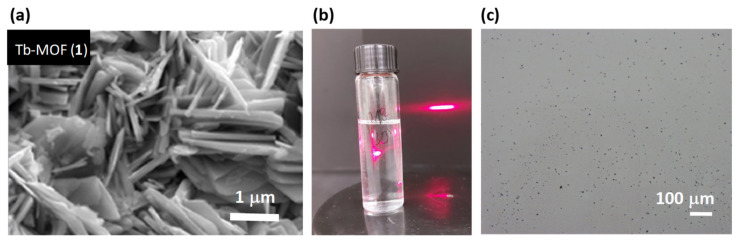
Exfoliation process from (**1**). (**a**) SEM image of crystals of **1** (bulk), (**b**) Tindall effect of a suspension of delaminated **1**, (**c**) flakes of **1** deposited onto a silicon wafer.

Figure 2a shows a SEM image of a bulk microcrystalline sample of **1**. Powder X-ray diffraction (PXRD) confirmed this bulk material is formed of microcrystals of **1**.

The presented material is composed of neutral 2D layers separated by weak Van der Waals interactions, and thus is ideal for exfoliation. In the past, several examples of coordination networks in two dimensions had been reported, usually relying on polytopic planar ligands or molecules [13,14,40]. However, in many cases, these 2D MOFs were formed of charged layers with the counterions in the lamellar space [41], which made them brittle and difficult to exfoliate. For exfoliation, neutral 2D MOFs, organized in three dimensions by weak Van der Waals forces are preferred, as the non-covalent bonding between layers allow the exfoliation without disturbing the bonding within the layer. We have calculated for the previously reported Dy analogue that the interaction energy between two interacting monolayers of the size of a unit cell is −113 kJ/mol (for comparison, the interaction energies of two-layer graphene and graphane, with the same surface size as our 2D MOF, are −274 and −97 kJ/mol, respectively) [39].

### 2.2. Exfoliation and Deposition of Tb-2D MOF Flakes onto Silicon

**Figure 3:**
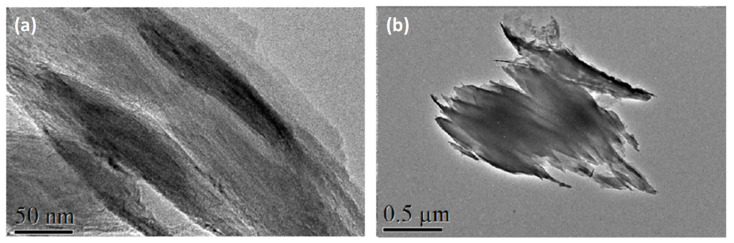
TEM images of exfoliated nanosheets on a carbon-copper grid.

Exfoliation of **1** into stable nanosheets was carried out by sonication of 1 mg of microcrystals in 5 mL of *i*-propanol. The mixture was sonicated in a VWR sonicator bath for 1 h at 30 °C. After this time, the dispersion was centrifuged at 4500 rpm for 10 min and the colorless dispersion of nanosheets decanted from the remaining white solid. The suspension of delaminated **1** produced the Tyndall effect (Figure 2b): when irradiating the colloidal solution of nanosheets the straight trajectory of the red laser is scattered in different directions. The *i*PrOH solution of nanosheets was stable for several months. Figure 3 shows TEM images of separate nanosheets of **1** deposited on a carbon Cu grid from the isopropanol solution. The nanosheets of **1** break easily upon sonication. Experiments are now being performed to obtain larger intact nanosheets using a shaker instead of ultrasounds. TEM shows that the nanosheets of **1** are broken, and form flakes of several nanosheets. In similar exfoliation conditions, for the Dy analogue, we were able to observe isolated nanosheets in TEM, as we reported [39].

**Figure 4:**
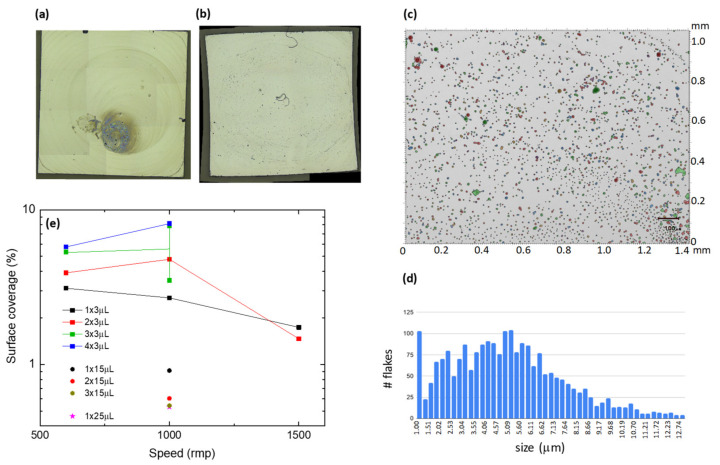
Optical image of 5 × 5 mm^2^ silicon wafer with deposited Tb-MOF flakes, by drop-casting (**a**) and spin-coating (**b**); (**c**) quantification of surface coverage and (**d**) distribution of flake sizes, for a spin-coated sample; (**e**) surface coverage determined for different spin-coating conditions.

The deposition of the exfoliated material onto a surface is a pre-requisite for further manipulation of the material and its application onto devices. Flakes of **1** material were deposited on a 5 × 5 mm^2^ silicon wafer by two methods: drop-casting and spin-coating. Different drop-casting experiments were performed, changing the number of droplets and volume of liquid. This method produced a non-homogeneous distribution of flakes in the center of the sample (see Figure 4a) and was, therefore, abandoned. In contrast, a uniform distribution of flakes was achieved by spin-coating (Figure 4b). Different experiments were performed by changing the drop volume, multiple deposition of drops, and rotation speed. The surface coverage was observed under an optical microscope and quantified with Mountains software (Figure 4c). Figure 4e summarizes the results of spin-coating experiments. A maximum coverage of ~8% could be achieved under optimized conditions. The histogram distribution of flake sizes shows that the average flake diameter is ca. 5 μm (Figure 4d).

**Figure 5:**
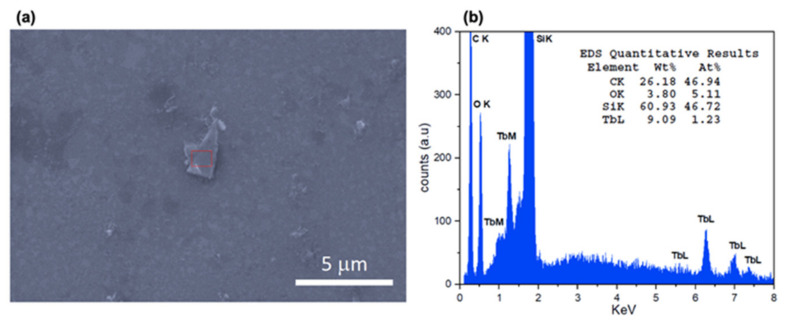
SEM image of a Tb-2D MOF flake deposited on silicon (**a**), and EDS analysis (**b**).

Figure 5a shows a SEM image of one of the deposited flakes on silicon. Energy dispersive X-ray spectroscopy (EDS) analysis on the flake confirms the presence of Tb (Figure 5b).

### 2.3. Luminescence and Raman Spectroscopy

**Figure 6:**
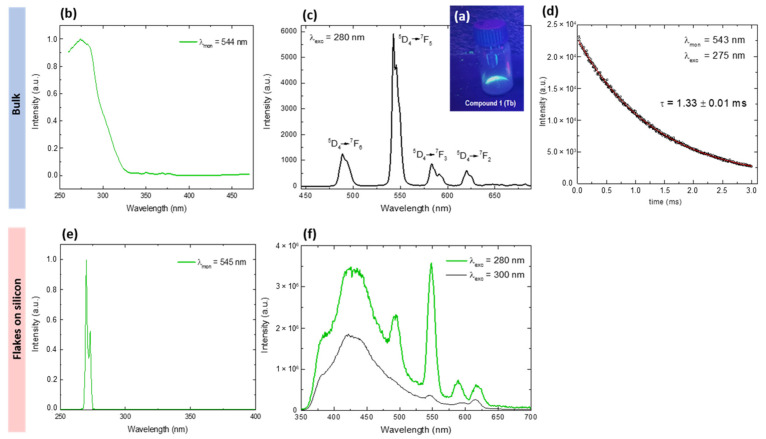
Top: luminescence of **1** in bulk: (**a**) Emission of powder under UV lamp, (**b**) excitation spectra monitored at λ_mon_ = 544 nm, (**c**) emission spectra upon ligand excitation at λ_exc_ = 280 nm, (**d**) lifetime measurement at λ_exc_ = 280 nm, monitoring the decay of the 544 nm peak, and fit to a single-logarithmic decay, with τ = 1.33 ms. Bottom: luminescence of flakes of **1** deposited on silicon: (**e**) excitation and (**f**) emission spectra.

First, the luminescent properties of **1** as bulk microcrystalline powder were characterized. When exposed to UV radiation the sample emitted green visible light (Figure 6a). The excitation spectrum, monitored at 544 nm, showed a broad band arising from the benzoate ligands at around 280 nm (Figure 6b); therefore, this wavelength was chosen for ulterior emission studies upon ligand excitation.

The emission spectrum of microcrystalline **1** exhibits four characteristic bands at 488, 546, 590 and 616 nm when excited at λ_exc_ = 280 nm. These correspond to the D45→FJ7 (J = 6, 5, 4, 3) transitions to the ground state multiplet of the Tb(III) ion (Figure 6c), dominated by the D45→F57 transition at 546 nm. The luminescence decay of this peak was τ_obs_ = 1.33 ± 0.01 ms (Figure 6d). The overall quantum yield upon excitation at 280 nm was found to be sizeable, $Q_{Tb}^{ligand}$ = 61 ± 1%.

The excitation and emission spectra for the flakes deposited on silicon are shown in Figure 6e,f, respectively. The emission spectrum recorded at λ_exc_ = 280 nm exhibits a broad band at low wavelengths, that stems from the silicon wafer reflection (indeed, the emission spectrum recorded for a pristine wafer presented the same band). However, above this background the characteristic D45→FJ7 emission peaks of Tb(III) are clearly observed (Figure 6f).

**Figure 7:**
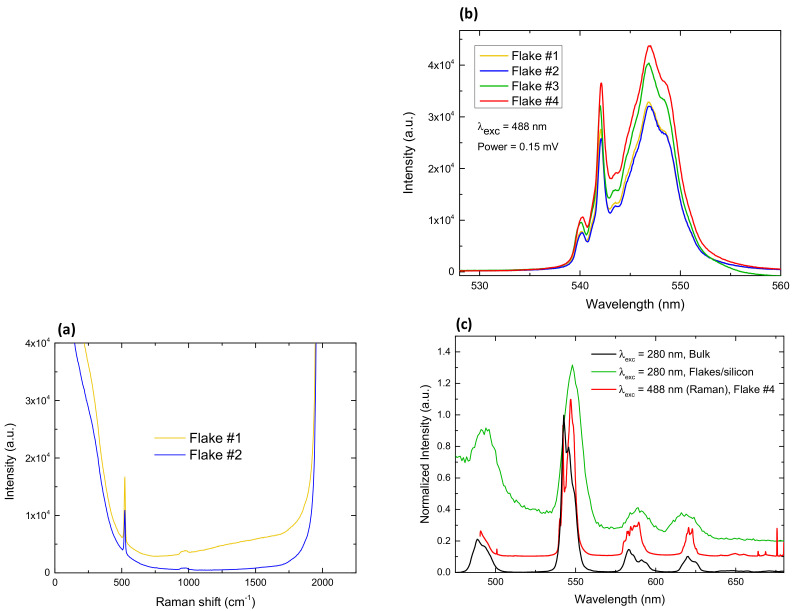
Raman spectroscopy of Tb-2D MOF flakes on silicon. (**a**) Raman shift spectra, relative to laser wavelength (488 nm) measured on two different flakes; (**b**) Tb emission spectrum peak measured on 4 different flakes, (**c**) Emission spectra obtained in Raman set up (λ_exc_ = 488 nm) from a single flake/silicon (red line) and of flakes on top of a Si waver upon ligand excitation, 280 nm (green line). Emission from bulk sample (black line). Spectra have been vertically shifted for the sake of comparison.

In addition, micro-Raman spectroscopy was used in order to probe the vibrational modes of Tb-MOF flakes. The setup was provided with a microscope allowing to focus the incident blue laser beam (448 nm) of power 0.15 mW onto individual flakes. The Raman shift spectra in the vibrational mode region (500–1745 cm^−1^) only exhibited the typical Raman peaks at 521 cm^−1^ and 965 cm^−1^ associated to the silicon wafer [42], as shown in Figure 7a. No other vibrational peaks associated with the flakes’ material could be distinguished, as the signal was dominated by the stronger tails of the Tb luminescence peaks in this experimental window.

Interestingly, the spatial resolution of our Raman setup allowed us to measure the luminescence spectra of individual flakes upon excitation at λ_exc_ = 488 nm (the laser wavelength). Figure 7b shows the 546 nm—Tb peak measured at four different flakes. The observed fine structure was very reproducible from one flake to another. Changes in the intensity of the peaks may be associated with the different thicknesses of the deposited flakes, or slight differences in the focusing. Figure 7c shows the full emission spectra recorded between 490–681 nm at one of the flakes. The position of the main peaks measured by Raman (red line) coincides with those measured by fluorimetry for the flakes-on-silicon (green line) and the bulk (black line), upon ligand excitation (280 nm). However, the emission spectrum obtained in the Raman set up shows further fine structure, due to the higher spectral resolution attained in the Raman system.

### 2.4. Magnetic Characterization

**Figure 8:**
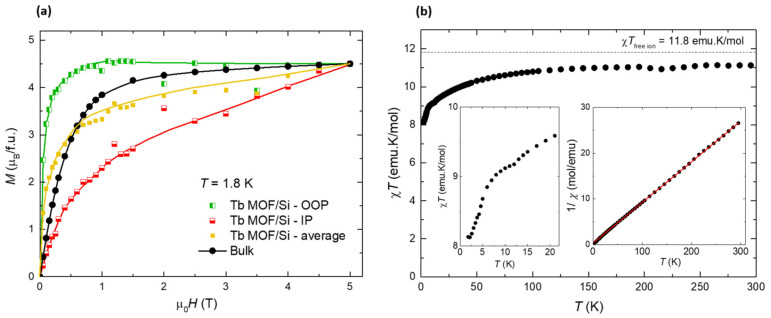
SQUID magnetic characterization of Tb-2D MOF of (**1**). (**a**) Field-dependence of the magnetization at *T* = 1.8 K for the bulk material, and flakes deposited on silicon, with the field applied in-plane (IP) and out-of-plane (OOP) with respect to the substrate, (**b**) temperature dependence of the susceptibility-temperature product, at *H* = 1 kOe, for the bulk. Inset: detail of the *χT*(*T*) curve at low-T; also, 1/*χ* curve and fit to a Curie-Weiss law.

The magnetization as a function of the applied field for a powdered sample of (**1**) is shown in Figure 8a. The *M*(*H*) curve measured at *T* = 1.8 K reaches for high fields, μ_0_*H* = 5 T, the expected saturation value for an angular random distribution of highly uniaxial anisotropic grains, *M*_sat_^powder^ = 0.5*g*_z_*S_z_* = 4.5 μ_B_/f.u., where *g*_z_* = 18 is the z-component of the gyromagnetic tensor for Tb within a S_z_* = 1/2 effective spin description. The magnetic susceptibility was investigated under 1 kOe in the range of 1.8–300 K and is shown as *χT*(*T*) plot in Figure 8b. The *χT* value at 300 K for **1** (11.16 emu K/mol) is slightly below the expected free-ion Tb^3+^ value. Upon cooling, *χT* decreases until reaching a plateau of 9.1 emu K/mol at 10 K, then decreases abruptly to a minimum value of 8.1 emu K/mol at 2.0 K, while an upturn in *χT* is hinted at lower temperatures (Figure 8b, inset). That decrease can be explained by the depopulation of the Tb crystal field (CF) split levels and the presence of AF interactions. The Curie–Weiss equation, *χ* = C/(*T* − θ), was used to fit the 1/*χ* (*T*) data, yielding C = 11.29 ± 0.07 emu K/mol and θ = −4.83 ± 0.01 K for **1**. The negative θ values indicate the existence of predominant antiferromagnetic (AF) dipolar interactions between the Tb ions. The magnetic behavior of the Tb-MOF compound qualitatively resembles that of the Dy-2D MOF analogue, previously reported [39], where all dipolar interactions between each Dy ion with its first neighbors were found to be AF. Although, the *χT* upturn, indicating the existence of competing ferromagnetic (FM) interactions at low temperatures, is less prominent in the former.

Static magnetic measurements were in addition performed on flakes deposited on a 5 × 5 mm^2^ silicon waver. The flakes’ magnetic contribution was obtained after carefully subtracting the substrate and holder contribution, separately measured. Figure 8a shows the *M*(*H*) magnetization curves, measured with the magnetic field applied in-plane (IP) and out-of-plane (OOP) with respect to the substrate. Since the absolute mass of the deposited flakes is unknown, the *M*(*H*) curve was normalized to match the saturation value of the bulk sample at 5 T. The results reflect the magnetic anisotropy of the nanosheets. The *M*(*H*) curve with the field OOP lies above the IP *M*(*H*) curve. The averaged curve, (*M*_IP_ + *M*_OOP_)/2, is close to the magnetization curve for the bulk, within the experimental uncertainty.

On the other hand, the experimental determination of the *χT*(*T*) for the deposited flakes was not possible, as their signal at high temperatures became too small compared to the substrate to allow a reliable substrate subtraction.

**Figure 9:**
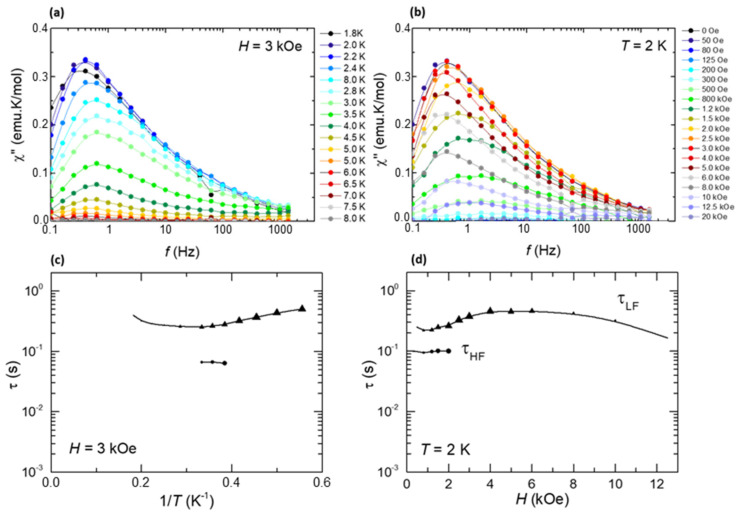
Top: out-of-phase component of the ac susceptibility as a function of the frequency, (**a**) at constant field *H* = 3 kOe and different temperatures, (**b**) at constant temperature *T* = 2 K and different applied fields for a powdered sample of (**1**). Bottom: magnetic relaxation time, (**c**) as a function of the inverse of the temperature, *τ*(1/*T*), at *H* = 3 kOe, and (**d**) as a function of the applied magnetic field, *τ*(*H*), at *T* = 2 K.

Alternating-current (ac) susceptibility measurements as a function of frequency (0.1–1 kHz), temperature (*T* = 1.8–8 K) and field (*H* = 0–30 kOe), were performed to characterize the relaxation properties of **1** in the bulk. The complex did not show frequency dependent out-of-phase *χ*” signal under *H* = 0. Upon application of a dc field, quantum tunneling of the magnetization (QTM) was effectively suppressed and slow relaxation of the magnetization effects was observed. The *χ*”(*f*, *T*) curves at optimum field *H* = 3 kOe, and *χ*”(*f*, *H*) data at *T* = 2 K are shown in Figure 9a,b. From the position of the *χ*” peaks, the relaxation time as a function of the inverse temperature and the magnetic field were determined (Figure 9c,d).

The intense peak *χ*” measured at low frequencies, shows relaxation time dependencies, *τ*_LF_(1/*T*) and *τ*_LF_(*H*), typical of a direct process mechanism affected by bottleneck. A second relaxational process is hinted at as a bump at higher frequencies; however, too few data are available to deduce the associated relaxation mechanism. Unlike in the Dy-2D MOF analogue [39], no clear thermally-activated process could be observed.

## 3. Experimental Methods

[Tb(MeCOO)(PhCOO)_2_] (**1**): the synthesis of [Tb(MeCOO)(PhCOO)_2_] (**1**) followed the procedure described in [39]. All chemicals and solvents were purchased from commercial sources and used as received. An amount of 0.0883 g of hydrated Tb(MeCOO)_3_·xH_2_O (0.26 mmol) and 0.063 g of PhCOOH (0.52 mmol) were placed in a CEM Discover Microwave Reactor with 4 mL of a MeOH:MeCN 1:1 mixture. A pulse of 150 W was applied and the reaction kept for 10 min at a maximum temperature of 125 °C. The reaction was cooled to room temperature and a colorless precipitate was filtered off. The solution was left for 9 days at 40 °C; after this time colorless crystals of **1** were obtained in 37% yield. The precipitate is analyzed by infrared (IR) and powder X-ray diffraction (PXRD) and found to be the same material. Total yield: 55%. Elemental analyses for **1**·H_2_O calculated (experimental): C 40.44 (40.79)%; H 2.55 (2.96)%. IR data (cm^−1^): 3069 (w), 3039 (w), 3023 (w), 1590 (s), 1536 (s), 1436 (w), 1394 (s), 1331 (s), 1248 (m), 1164 (s), 1068 (w), 1022 (w), 993 (m), 930 (m), 868 (w), 10.1002/anie. 847 (w), 830 (w), 788 (m), 709 (s), 700 (s), 687(s), 667 (s), 617 (w), 604 (w).

Exfoliation: 1 mg of **1** is placed in a vial with 5 mL of *i*-PrOH. The tube is placed in a VWR sonicator at 45 KHz and 120 W with a water bath at 30 °C for 1 h. The remaining solid is separated by decantation after centrifugation (4500 rpm, 10 min) to separate the suspension of nanosheets. The dispersion of nanosheets displays Tyndall effect and is stable for at least 3 months.

X-ray diffraction data for **1** were collected with a Bruker APEXII SMART diffractometer using a Molybdenum K-alfa microfocus radiation source (lambda = 0.71073 Å) at GMMF, and only a unit cell was obtained. Samples were taken to synchrotron source Advanced Light Source, beamline 12.2.1 (Berkeley, CA, USA) and collected using shutterless phi and omega scans on a Bruker D8 with PHOTON II detector at 0.7288 nm. The structures were solved by Patterson or intrinsic phasing methods (SHELXS2013 and SHELXT) and refined on F_2_(SHELXL-2016). Hydrogen atoms were included on calculated positions, riding on their carrier atoms. Powder X-ray diffraction data for **1** (precipitate), and **1** (crystals) were collected at the *Centres Científics i Tecnològics-Universitat de Barcelona* (CCiTUB).

Crystal data and some further details concerning x-ray analysis are given in Table 1. The bond lengths and angles for 1 are listed in CCDC-2095560. These data can be obtained free of charge via www.ccdc.cam.ac.uk/conts/retrieving.html (or from the Cambridge Crystallographic Data Centre, 12 Union Road, Cambridge CB2 1EZ, UK; fax: (+44) 1223-336-033; or deposit@ccdc.ca.ac.uk).

Scanning Electron Microscopy (SEM): the bulk specimen was coated in graphite and analyzed on the CCiTUB facilities. SEM images and EDS of flakes deposited onto silicon were taken without C coating at ICMAB facilities.

Static magnetic measurements: magnetization, *M*(*H*), and susceptibility, *χ*(*T*), data from powdered polycrystalline samples were measured with the addition of Daphne oil to avoid grain orientation, in a Quantum Design SQUID Magnetometer equipped at Universidad de Zaragoza with the RSO option and a Quantum Design PPMS with the VSM option. *M*(*H*) was measured at *T* = 1.8 K up to 140 kOe and *χ*(*T*) from 1.8 to 300 K, with an applied field of 1 kOe.

Dynamic magnetic measurements for polycrystalline samples were conducted at fixed temperatures in the range 1.8 < *T* < 6 K, with an excitation field of 4 Oe, at dc bias fields in the range 0 < *H* < 50 kOe, while sweeping the frequency 0.1 < *f* < 1000 Hz in the same SQUID magnetometer.

*M*(*H*) at 1.8 K from 0–5 T, and *χ*(*T*) at 1 kOe from 1.8–300 K curves of flakes deposited onto 5 × 5 mm^2^ silicon wafers, with the field *H* applied IP and OOP with respect to the substrate, were measured in a SQUID magnetometer (MPMS3 from Quantum Design) in DC scan at ICMAB. The pristine substrate was measured, with the field applied in the two directions, so as to subtract the diamagnetic contribution.

Photoluminescence emission and excitation spectra of **1** (bulk) were measured using a Fluorolog FL-1057 Jobin Ybon HORIBA spectrofluorometer. Phosphorescence lifetimes were recorded with a Fluoromax phosphorimeter accessory containing a UV xenon flash tube. Quantum yield (QY) was measured using a Hamamatsu Absolute PL Quantum Yield spectrometer C11347 (Hamamatsu Quantaurus QY). The absolute QY (ratio of the number of photons emitted by photoluminescence to the number of photons absorbed by the light-emitting material) was measured using an integrating sphere. Powdered samples were placed in a capillary with an internal diameter of 1 mm. The absorption and emission spectra of the sample container (the blank) were separately recorded. The QY was calculated as: $Q=(E_{c}-E_{a})/(L_{a}-L_{c})$ with *E*_c_ being the integrated emission spectrum of the sample, *E*_a_ being the integrated blank emission spectrum, *L*_a_ being the blank absorption, and *L*_c_ being the sample adsorption at the excitation wavelength.

Luminescence studies of flakes of **1** on silicon wafers were measured using the spectrofluorometer NanologTM-Horiba Jobyn Yvon fluorimeter at the Inorganic Chemistry Section, UB. The excitation and emission spectra of the deposited flakes on a silicon wafer were measured in the fluorimeter in a “Right Angle” configuration, i.e., with the incident and sensed beams forming 90°, and the incident light impinging the sample at 45°.

Raman scattering experiments were performed at room temperature in the backscattering geometry using a T64000 Horiba Jobin-Yvon micro-Raman spectrometer equipped with a high sensitivity liquid nitrogen cooled CCD (charge-coupled device) as the detector, and an Olympus BX40 microscope. The 488 nm laser line was used for the measurements. Samples were mounted on the rotation X-Y stage of the microscope [43]. The incident laser beam was focused to a ≈1 μm spot using a ×100 microscope objective on the sample. The laser power was kept below 0.15 mW to avoid laser-induced heating and degradation of the analyzed flakes. The spectrometer resolution was 2 cm^−1^.

## 4. Conclusions

A simple, microwave assisted method was used for the synthesis of a new magnetic and luminescent Tb 2D-MOF material (**1**). Compound **1** can be easily delaminated in an isopropanol solution by sonication. A uniform distribution of 5 μm-size flakes, covering up to an 8% of a 5 × 5 mm^2^ silicon substrate could be prepared by spin-coating. The deposited Tb-2D MOF flakes exhibit both luminescence in the visible, and axial magnetic anisotropy. Hence, our method represents a promising approach for the preparation of multifunctional 2D materials on different surfaces, allowing for the development of hybrid materials, multilayer hetero-nanostructures or devices for different applications.

## Figures and Tables

**Table 1:** Single crystal X-ray diffraction data for compound **1**.

**Formula**	[Tb(MeCOO)(PhCOO)_2_] (**1**)
**CCDC**	2095560
**Crystal System**	Monoclinic
**Space Group**	P2_1_/c
**Z**	4
**a (**Å**)**	16.3617(9)
**b (**Å**)**	12.6659(7)
**c (**Å**)**	7.2496(4)
**Alfa^o^**	90
**Beta^o^**	101.073(2)
**Gamma^o^**	90
**V (**Å**^3^)**	1474.41(14)
**T (K)**	100(2)
**X-ray**	ALS
**R (wR)**	0.0258 (0.0563)

## Data Availability

Not applicable.

## Sample Availability

Samples of the compounds are not available from the authors.
